# Perception of care in immigrant and nonimmigrant persons with inflammatory bowel disease: a survey-based study

**DOI:** 10.1093/jcag/gwag013

**Published:** 2026-04-17

**Authors:** Christiane Gafrey, Alexa Sasson, Maria Cino, Girish Bajaj, Thurarshen Jeyalingam, Parul Tandon

**Affiliations:** Department of Medicine, University of Toronto, Toronto, Ontario, M5S 3H2, Canada; Department of Medicine, University of Toronto, Toronto, Ontario, M5S 3H2, Canada; Division of Gastroenterology and Hepatology, University Health Network, Toronto, Ontario, M5G 2C4, Canada; Department of Medicine, University of Toronto, Toronto, Ontario, M5S 3H2, Canada; Division of Gastroenterology and Hepatology, University Health Network, Toronto, Ontario, M5G 2C4, Canada; Department of Medicine, University of Toronto, Toronto, Ontario, M5S 3H2, Canada; Department of Medicine, University of Toronto, Toronto, Ontario, M5S 3H2, Canada; Division of Gastroenterology and Hepatology, University Health Network, Toronto, Ontario, M5G 2C4, Canada; Department of Medicine, University of Toronto, Toronto, Ontario, M5S 3H2, Canada; Division of Gastroenterology and Hepatology, University Health Network, Toronto, Ontario, M5G 2C4, Canada

**Keywords:** immigrant, healthcare, inflammatory bowel disease (IBD)

## Abstract

**Background and Aims:**

Immigrants are at a heightened risk of developing inflammatory bowel disease (IBD) after relocating, and their interactions with the healthcare system differ from those of nonimmigrants. This study aimed to explore the challenges and perspectives of immigrants living with IBD regarding their healthcare.

**Methods:**

We recruited persons aged 18 or over with IBD, including those with and without an immigration history, to complete an anonymous online survey. Descriptive statistics were presented as frequencies, and cross-group comparisons were performed using the Chi-square test.

**Results:**

We included 75 immigrants and 150 nonimmigrants with similar baseline demographics, with many indicating poorly controlled IBD. Immigrants were more likely to report no access to formal translation services during their appointments (24 [32%] vs 6 [8%]), despite many not speaking the same language as their gastroenterologist (31 [41.3%] vs 3 [2%]). Immigrants were more often unable to miss work for healthcare appointments (24 [32%] vs 17 [11.3%]). Many were diagnosed after immigration (58 [77.3%]). Despite this, 29 [38.7%] contacted physicians in their home countries and purchased medications from their countries of origin (8 [10.7%]). Most immigrants expressed concerns that “Western” foods in their current country of residence contributed to their flares (44 [58.7%]) compared to only 11 [14.7%] who were concerned about foods from their home country.

**Conclusions:**

Immigrants encounter significant obstacles in accessing healthcare, including communication, financial barriers, and cultural factors such as food preferences, which hinder their ability to receive the care they need.

## Introduction

Inflammatory bowel disease (IBD) is a chronic condition affecting individuals of all ages.^1^ There are 2 main subtypes—ulcerative colitis (UC) and Crohn’s disease (CD). The prevalence of IBD is increasing globally,^2^ highlighting the need for understanding the nature of this disease and its effect on public health. It has been previously established that access to high-quality, equitable care for all individuals with IBD may lead to improved long-term disease and patient-related outcomes.^3^ Despite this, certain individuals with IBD, such as immigrant persons to new healthcare settings, may be vulnerable to inequalities in healthcare access, potentially resulting in poor long-term outcomes.^3^

Previous studies have reported that individuals who migrate from a lower-income country to a higher-income country have a higher incidence of IBD.^4^ Additionally, a lower age at the time of immigration increases the risk of developing IBD.^5^ Immigrants with IBD may also have distinct disease patterns compared to native residents, which may result in different healthcare utilization trends.^6^ Furthermore, certain groups with IBD may be more likely to experience disease-related complications. For example, South Asian individuals with IBD may be more likely to have fistulizing CD and pan-UC,^6^ whereas black immigrants may be more likely to undergo CD-related surgery.^4^ Immigrant individuals also have greater IBD-specific outpatient health services use after diagnosis (odds ratio [OR] 1.24; 95% CI, 1.15-1.33), emergency department visits (OR 1.57, 95% CI, 1.30-1.91), and hospitalizations (OR 1.19; 95% CI, 1.02-1.40).^7^

Despite these findings, there is limited understanding of the specific factors driving these differences in healthcare utilization, such as potential barriers to access to equitable care. These barriers may lead to inefficient use of healthcare resources and suboptimal care for this population. Immigrant persons often present with complex health needs, influenced by a range of confounding factors, such as limited prior access to healthcare, language barriers, and potentially lower levels of health literacy.^8^ Each of these factors necessitates more nuanced and culturally competent care. However, there remains a significant gap in understanding how to effectively tailor the management of IBD for this population.

As such, this study aimed to explore how immigrants perceive their IBD care in comparison to nonimmigrant persons. Understanding these factors may then lead to interventions that may improve patient outcomes and identify opportunities for optimizing care delivery within this diverse population.

## Methods

This study was approved by the research ethics board at the University Health Network (UHN) in Toronto, Ontario, Canada (REB: 24-5262.1). All data remained anonymous throughout the study.

### Study design and recruitment

An online questionnaire was developed in English, to evaluate the perceptions regarding healthcare for individuals with IBD among both immigrant and nonimmigrant persons. The inclusion criteria for participation included age 16 years or older, self-reported diagnosis of IBD, and ability to determine immigration status based on self-reported answers. An immigrant was defined as anyone born outside of the country where they are currently residing. Recruitment occurred from July 1, 2024, to July 1, 2025, with all participants residing in North America. Participants were recruited from social media platforms such as Reddit, X (formerly Twitter), and Instagram, as well as physical posters displayed in clinical areas at the UHN hospitals (Toronto General Hospital and Toronto Western Hospital). Due to the pilot nature of this study, it was *a priori* determined to recruit up to 75 and 150 nonimmigrant participants with IBD.

### Study outcomes

The primary outcome of this study was the comparison of healthcare access and perceived barriers to care, including financial, literacy, and communication factors, between immigrants and nonimmigrants with IBD. Secondary outcomes included comparison of the above differences of the above between “Western” and “non-Western” patients, in addition to individual regions of origin based on the World Health Organization classifications.^9^ “Western” immigrant participants were those classified in North America (excluding Mexico) and Europe, with all other immigrant participants in the “non-Western” category.

### Survey content

Several baseline questionnaires were provided to all participants to complete (see Document S1 for full survey). These included the demographic questionnaire (age, sex, gender identity, primary language, relationship status, employment status, and education level), immigration questionnaire (immigration history), and an IBD questionnaire (subtype, treatment, stool frequency, rectal bleeding, presence of urgency, prior hospitalizations, and steroid use). All participants were then asked to provide information regarding their IBD-related healthcare experiences. This included questions about their understanding of medical appointments and subsequent management plans, their ability to communicate with their healthcare providers, the nature of recent visits (virtual or in-person appointments), access to translation services and to supportive resources such as nurses trained in IBD or educational materials. Additionally, participants were asked about financial concerns, such as the ability to afford to miss work, attend appointments, or pay for medications necessary for managing their IBD.

Those who reported an immigration history were also asked to complete immigration-specific questions such as country of origin, the country they immigrated to, the year of immigration, access to traditional foods compared to those available in their new country, healthcare coverage, medication accessibility and affordability, and access to gastroenterologists and IBD-related appointments.

Finally, physician trust was assessed using the validated Trust in Physician Survey (TPS)^10^ and the impact of IBD on work productivity by the validated Work Productivity and Activity Impairment Questionnaire (WPAIQ).^11^ The TPS is an evidence-based tool tested in numerous populations of patients, including differing racial and ethnic populations. It consists of 11 items rated on a 5-point Likert scale with answers ranging from strongly agree to strongly disagree. Scores can range from 11 to 55 with the higher numbers indicating a strong level of trust.^10^ The WPAIQ includes 6 questions which measure work-related absenteeism, presenteeism, overall work productivity loss, and general activity impairment. A score of 0 indicates no effect of the chronic disease on work or daily activities and 10 indicates complete impairment. Higher overall scores suggest a greater level of impairment.^11^

### Statistical analysis

Descriptive statistics were used to assess baseline characteristics between immigrant and nonimmigrant groups. Continuous variables were summarized using medians and interquartile ranges (IQRs), while categorical variables were presented as frequencies and percentages.

Responses from the TPS and the WPAIQ were analyzed using median scores with IQRs, given the distribution of data. Contingency tables were constructed to compare categorical responses between groups. Group comparisons for categorical variables were performed using Chi-square tests or Fisher’s exact tests where appropriate. Furthermore, subgroup analysis was completed using the methods detailed for “Western” versus “non-Western” groups. Open-ended survey responses were analyzed thematically according to patient experience and barriers to care. All statistical analyses were performed using IBM SPSS Statistics (Version 29). As this is a pilot hypothesis-generating study, only observed numerical differences were described.

## Results

### Baseline demographics

We included 75 immigrant and 150 nonimmigrant participants with IBD. The median age at time of survey completion was 38.5 (IQR 17) and 42.0 (IQR 25) for immigrants and nonimmigrants with IBD, respectively. There were no differences in sex, gender identity, relationship status, employment status, and educational between immigrants and nonimmigrants with IBD (Table 1).

**Table 1:** Baseline characteristics.

Patient characteristics	Immigrant (*n* = 75)	Nonimmigrant (*n* = 150)
**Age at survey completion, median (IQR)**	38.5 (IQR 17)	42.0 (IQR 25)
**Sex, *n* (%)**		
** Male**	35 (46.7)	51 (34)
** Female**	40 (53.3)	99 (66)
**Gender identity, *n* (%)**		
** Man**	35 (46.7)	50 (33.3)
** Woman**	40 (53.3)	97 (64.7)
** Nonbinary**	-	2 (1.3)
** Transgender**	-	1 (0.7)
**Relationship status, *n* (%)**		
** Common law**	6 (8)	15 (10)
** Divorced**	1 (1.3)	4 (2.7)
** Married**	43 (57.3)	65 (43.3)
** Prefer not to say**	1 (1.3)	3 (2.0)
** Separated**	0 (0)	2 (1.3)
** Single**	23 (30.7)	57 (38)
** Widowed**	1 (1.3)	4 (2.7)
**Employment status, *n* (%)**		
** Full time employed**	48 (64)	84 (56)
** Part time employed**	6 (8)	12 (8)
** Home maker**	4 (5.3)	4 (2.7)
** Looking for work**	5 (6.7)	6 (4)
** Other**	4 (5.3)	20 (13.3)
** Sick leave**	1 (1.3)	2 (1.3)
** Student**	4 (5.3)	18 (12)
** On disability due to GI illness**	3 (4)	8 (5.3)
**Education level, *n* (%)**		
** College**	14 (18.7)	24 (16)
** Doctoral degree**	7 (9.3)	9 (6)
** High school graduate**	11 (14.7)	21 (14)
** High school incomplete**	1 (1.3)	0 (0)
** Master’s degree**	14 (18.7)	34 (22.7)
** Bachelor’s degree**	27 (36)	60 (40)

Abbreviation: GI, gastrointestinal.

### Immigrants with IBD

Of the 75 immigrants with IBD, their birth region was Africa/Caribbean in 7 (9.3%), North America in 4 (5.3%), Asia in 5(6.6%), Europe in 19 (25.3%), Middle East in 12 (16%), South Asia in 24 (32%), and South America in 4 (5.3%). Seventy-three individuals (97.3%) resided in Canada at the time of the survey completion. Sixteen (21.3%) immigrants were diagnosed with IBD prior to immigration, 58 (77.3%) after immigration, with a median time from immigration to diagnosis in the latter of 10 years (IQR 12).

### IBD characteristics

There were no significant differences in IBD characteristics between the 2 groups (Table 2). Specifically, 41 (54.7%) and 60 (40.0%) of immigrants and nonimmigrants, respectively, reported having a diagnosis of UC (Table 2). Most immigrants and nonimmigrant participants reported being corticosteroid-free in the 7 days prior to survey completion. Many immigrants and nonimmigrants with IBD reported having abdominal pain (49.3% vs 59.3%), urgency (62.6% vs 69.0%), and being generally unwell in the 7 days before survey completion (53.0% vs 52.0%). There were no differences in emergency department visits or hospitalizations between the 2 groups (Table 2).

**Table 2:** Inflammatory bowel disease (IBD) characteristics.

IBD characteristics	Immigrant (*n* = 75)	Nonimmigrant (*n* = 150)
**IBD subtype, *n* (%)**		
** Crohn’s disease**	23 (30.7)	60 (40)
** Ulcerative colitis**	41 (54.7)	60 (40)
** Unclassified**	6 (8)	10 (6.7)
** Unsure**	4 (5.3)	19 (12.7)
**Steroid use (past 7 days), *n* (%)**		
** Yes**	9 (12)	14 (9.3)
** No**	66 (88)	136 (90.7)
**Symptoms and clinical features, *n* (%)**		
** Abdominal pain**	37 (49.3)	89 (59.3)
** Rectal bleeding**	23 (30.6)	42 (28)
** Urgency**	47 (62.6)	104 (69)
** Bowel movements per day, median (IQR)**	2 (2)	2 (2)
** Liquid stools per day, median (IQR)**	0 (2)	0 (1)
** General wellbeing (“Under Par” or “Poor”)**	40 (53)	78 (52)
**Emergency department visit (past 6 months), *n* (%)**	17 (22.7)	18 (12)
**Hospitalization (past 6 months), *n* (%)**	6 (8)	10 (6.7)

### Communication barriers

In total, 142 nonimmigrant respondents (94.7%) reported English as their primary language, compared to only 36 (48.0%) of immigrant respondents. Notably, 124 (82.7%) nonimmigrant participants spoke the same language as their gastrointestinal (GI) doctor compared to only 36 (48%) of immigrants. Only 8 (6%) immigrants reported having access to formal translational resources at their gastroenterology visit. There appeared to be no significant differences in the understanding of their disease or treatment plan.

### Healthcare appointments and access to care

Most immigrant participants were covered under the Ontario Health Insurance Plan (90.7%), which represents universal health insurance. Forty-eight (64%) immigrant respondents did not have access to a dedicated IBD nurse compared to 63 (42%) of nonimmigrants. Many immigrants and nonimmigrants felt comfortable talking to their physician about their IBD symptoms (85.3% vs 83.3%). There were no differences in the proportion of patients undergoing predominant virtual care follow-up with their gastroenterologist (24, 32% vs 54, 36%) or reporting a lack of access to a family doctor (18, 25% vs 19, 12.7%) between immigrants and nonimmigrants, respectively.

### Financial concerns

**Figure 1:**
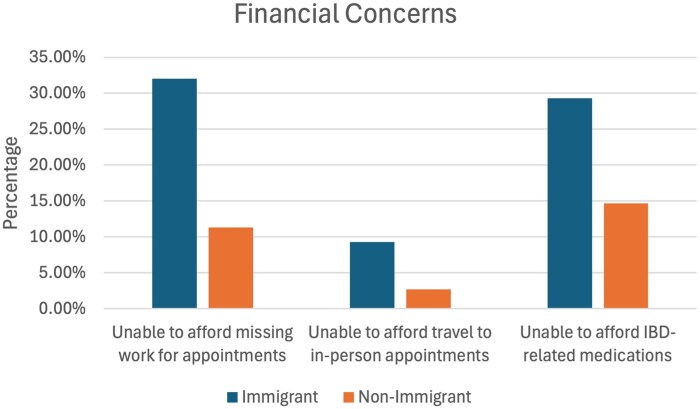
Financial concerns of immigrants and nonimmigrants with IBD.

Compared to nonimmigrants, immigrant participants were more likely to report being unable to afford missing work for medical appointments (24 [32.0%] vs 17 [11.3%]). However, there were no differences in being unable to afford travel costs to attend in-person appointments (7 [9.3%] vs 4 [2.7%]) or being unable to afford IBD-related medications (22 [29.3%] vs 22 [14.7%]) between the 2 groups (Figure 1).

### Cultural considerations

Most immigrants were diagnosed after immigration (58 [77.3%]). Despite this, 29 (38.7%) immigrant participants with IBD had contacted gastroenterologists in the countries they migrated from, and 8 (10%) bought IBD medicine from these countries, despite the majority being diagnosed with IBD after immigration. Of the immigrant participants who contacted a gastroenterologist in their home country, 24.1% were diagnosed with IBD before immigration. Additionally, most immigrant participants (44, 58.7%) had concerns that “Western” foods would negatively impact their IBD, whereas only 11 (14.7%) had concerns about their home food.

### Trust in physicians

When assessing the TPS scores, the median score was 35 (IQR 5) and 36 (IQR 5) in immigrants and nonimmigrants, respectively.

### Work productivity and impairment

Fifty-eight (77.3%) immigrants were employed for pay, compared to 101 (67.3%) nonimmigrants. On average, employed immigrant participants missed more work than nonimmigrant participants for their IBD. This is in the context of nonimmigrant participants working 40 hours (IQR 8), and immigrant participants working 40 (IQR 15) hours per week. On average, productivity impairment was minimal for nonimmigrants 2 (IQR 4) and 3 (IQR 6) immigrant participants. Similarly, IBD impairing regular activities was also minimal between the 2 groups, 2 (5 IQR) and the immigrant group, 3 (IQR 5).

### Differences across subgroups in immigrants with IBD

There were differences between Western and non-Western immigrants, with more non-Western immigrant participants being unable to afford missing work (36.5% vs 21.7%), and being unable to travel to in-person appointments (11.5% vs 4.3%). Western immigrants were more likely to be concerned about food, buy medicine in the country they migrated from, and contact their GI doctors from their country prior to migration (Table 3).

**Table 3:** Western versus non-Western immigrants.

	Western immigrants, *n* (%) (*n* = 23)	Non-Western immigrants, *n* (%) (*n* = 52)
**Ulcerative colitis**	11 (47.8)	30 (57.7)
**Crohn’s disease**	10 (43.5)	13 (25)
**GI does not speak their language**	10 (43.5)	21(40.4)
**No formal translation materials available**	8 (34.8)	16 (30.8)
**IBD nurse access**	6 (26.1)	9 (17.3)
**TPS, median (IQR)**	36 (IQR 5)	36 (IQR 4)
**Unable to afford missing work for appointments**	5 (21.7)	19 (36.5)
**Unable to afford travel to in-person appointments**	1 (4.3)	6 (11.5)
**Unable to afford IBD-related medications**	8 (34.8)	14 (26.9)
**Bought medicine back home**	3 (13)	5 (9.6)
**Contacted GI in home country**	11 (47.8)	18 (34.6)
**Home food concerns**	3 (13)	8 (15.4)
**Western food concerns**	16 (69.6)	28 (53.8)

Abbreviations: IBD, inflammatory bowel disease; GI, gastrointestinal; TPS, Trust in Physician Survey.

When assessing specific geographical regions of origin, respondents from Asia (excluding South Asia) were the most affected by language barriers, with 60% lacking access to formal translation services and 60% reporting that their gastroenterologist did not speak their primary language. Among South Asian respondents, 50% reported being unable to miss work for medical appointments, a higher proportion than in other groups (Table S4).

## Discussion

Individuals who migrate from low-income to high-income countries are at a significant risk of developing IBD.^12^ Moreover, immigrants with chronic diseases such as IBD may engage with the healthcare system differently compared to nonimmigrant populations.^7^ Our data suggest significant disparities in care, highlighting notable barriers and differences in how immigrant and nonimmigrant patients perceive and manage their IBD, with the most noticeable differences found in language and communication difficulties, financial constraints, limited utilization or access to healthcare services, and cultural issues that impact their care experience. Further research would need to be done to ascertain why these barriers exist.

Previous work has demonstrated that language discordance between patients and healthcare professionals can negatively impact health outcomes, patient satisfaction, and the overall quality of care.^13^^,^^14^ While it is difficult to ascertain why, language barriers were evident in our data. Only half of immigrant respondents reported English as their first language, and a majority did not speak the same language as their gastroenterologist. Furthermore, very few reported any access to any formal translation. Within the immigrant population, those migrating from non-Western countries were much less likely to report a lack of access to translation materials. As the study was conducted in English, we assume that all immigrant participants were able to read and write in English at a basic level, which may introduce bias in assessing access to translation, as these services may not have been offered or perceived as necessary.

Despite this, although immigrant participants may maintain a positive therapeutic relationship with their providers, communication barriers may limit the depth and effectiveness of these relationships as well as contribute to poorer outcomes.^15^ Furthermore, in chronic diseases such as IBD, nuances around disease management may not be as clearly communicated, particularly in severe disease states. This can lead to poor medication adherence, poor comprehension of instructions, and increased support required.^15^ Addressing linguistic needs through formal translation services and language-concordance may strengthen the provider-patient relationship, but this needs to be confirmed in future studies.

A significant proportion of immigrant respondents with IBD reported being unable to afford missing work to attend medical appointments, highlighting potential financial strain and limited job flexibility. Furthermore, immigrants from non-Western countries were less likely to afford missing work for appointments. This is consistent with previous work that has demonstrated that although immigrant persons are often employed, they tend to occupy lower wage jobs with minimal flexibility and coverage.^16^ The increase in availability of virtual care may reduce barriers to access to care that are due to travel, income loss, and missed workdays.^16^ Fortunately, in our study, a majority of individuals, immigrant and nonimmigrant, reported having adequate access to virtual care for their IBD. Beyond educational resources, financial barriers remain a significant challenge for many patients, particularly non-Western individuals, even when healthcare coverage is available. Costs primarily associated with logistics, including transportation and time off work, can impact a patient’s ability to seek and maintain care, further emphasizing the need for accessible and flexible healthcare options. These findings raise important questions about optimizing healthcare delivery for these populations. Continuing virtual visits can improve logistical barriers while ensuring continued patient engagement with healthcare providers, as well as improve satisfaction, as reported previously.^17^

A significant proportion of immigrant respondents in our study also reported seeking care for their IBD in countries that they migrated from.^17^ “Medical tourism” is a well-documented phenomenon demonstrated previously.^18^ Many immigrant individuals may seek care when returning to their host country, as this can be associated with lower cost of medical care and perhaps more enhanced social connections.^17^ Furthermore, increased familiarity with healthcare providers of similar culture and ethnic background may allow for more comfort in discussing more nuanced conversations around intimate questions.^19^ Cultural discordances between patients and their healthcare providers may lead to decreased comfort levels and poor health outcomes.^19^ Subsequently, physicians trained with cross-cultural competence may provide a better quality of care as perceived by patients.^20^ Overall, there are nuances to why patients return to their home countries for care, but Canadian practitioners should be aware of this phenomenon, particularly when developing management plans or initiating new medications.

Multidisciplinary care can improve the management of complex diseases, leading to a reduction in disease flares through preventive care, particularly in IBD.^21^ Particularly, the collaboration between nurses specialized in IBD, general practitioners, physician specialists, dieticians, and social workers may improve patient self-efficacy^22^ and reduce disease burden.^23^ However, many participants in our study lacked access to a dedicated IBD nurse, regular primary care support, and nutritional support. Consequently, many perceived the “Western” diet as strongly implicated in the development of their IBD.^20^ The lack of access to an IBD nurse may contribute to these barriers, as IBD nurses often play key roles in patient education and supportive management. Our data suggesting poor access to IBD nurses and dietary advice are in keeping with prior studies.^24^ Specifically, when surveying physicians and other healthcare providers, approximately half were not incorporating an integrated care model despite the understanding that nurses, psychologists, and dieticians are part of a successful IBD treatment.^24^ Moreover, the observed lack of family physician access among immigrants also represents a barrier to specialist care, given the referral-based nature of the Canadian healthcare system. While the lack of multidisciplinary care may be explained by patients’ underutilization or not knowing this service exists, we believe that our findings still highlight a need that providers can address.

Despite the findings detailed above, limitations do exist. Our study included a large number of immigrant and nonimmigrant respondents with IBD who were able to respond anonymously, thereby limiting, but not eliminating, selection bias. We also note that this is not a random sample of IBD patients, as approximately half of the participants reported abdominal pain, urgency, and generally feeling unwell, with more care inadequacies likely to be perceived while patients remain unwell. Given that most respondents were from Canada, our results may not be generalizable to immigrants in other countries. Furthermore, given the questionnaire-based methodology, the experiences shared by immigrant participants may not fully represent those of nonparticipants or other immigrant populations not included in the study. Additionally, since the study was conducted online and in English, certain groups, such as individuals without internet access or electronic devices, as well as those not proficient in English, may have been underrepresented or unable to participate. While this study succeeded in identifying how immigrant participants with IBD perceive their care, we are unable to determine why this is the case with certainty and this would require further research. Confounders are present when exploring why immigrant participants were returning to their home country, which could be related to familiarity with their home country’s healthcare system or lack of trust in the North American system. This was not assessed in this study. Similarly, when assessing access to an IBD nurse or a dietitian, it is unclear whether participants did not use these services because they were not offered, or because they were unaware of these services. Although we have identified areas that need to be addressed, the underlying causes and the subsequent means of addressing them require further study.

The views reported in this study were cross-sectional; changes in perceptions of care with increasing duration in the destination country should be examined in a future study. Additionally, further research should examine the drivers of immigration, household income, financial strains such as debt and family obligations, and how access to healthcare and its costs contribute to these financial burdens. Furthermore, a more in-depth thematic approach should be done using focus groups to explore further the immigrant perspective on how to address the barriers they face when accessing health care.

Overall, our study revealed that immigrant persons reported limited access to both basic and translational healthcare services, identifying a significant communication barrier, and financial pressures that impacted their ability to attend appointments. Additionally, they faced the complex task of managing their disease within a new cultural environment, expressing concern over unfamiliar foods and lifestyle changes that added to challenges in their disease management. Essential interventions include expanded access to professional translation services, culturally informed dietary guidance, and increased flexibility in appointment scheduling, such as offering virtual consultations and varied time slots. In summary, these findings represent a critical first step toward delivering culturally responsive and equitable care for immigrant patients with IBD.

## Supplementary Material

gwag013_Supplementary_Data

## Data Availability

The data underlying this article are available in the article and in its online supplementary material.
