# Supplementary material for: Perception of care in immigrant and nonimmigrant persons with inflammatory bowel disease: a survey-based study
Source: J Can Assoc Gastroenterol. 2026 Apr 17;9(4):225–31. doi: 10.1093/jcag/gwag013 (PMC13436814; doi:10.1093/jcag/gwag013)
Supplement: Supplementary file 1 [file gwag013_supplementary_data.zip › Supplemental Materials.docx]

Supplemental Materials

|  | **Afro-Caribbean (n=7)** | **Asia (n=5)** | **Europe**  **(n=19)** | **Middle East (n=12)** | **North America**  **(n=4)** | **South Asia**  **(n=24)** | **South America (n=4)** | **p-value** |
| --- | --- | --- | --- | --- | --- | --- | --- | --- |
| **GI does not speak their language** | (14.3%) | 60% | 52.6% | 50% | 0% | 37.5% | 50% | <0.001 |
| **No formal Translation Materials Available** | (14.3%) | 60% | 42.1% | 33.3% | No answer | 29.2% | 25% | <0.001 |
| **Virtual appointments** | (14.3%) | 0% | 42.1% | 33.3% | 25% | 33.3% | 50% | 0.598 |
| **IBD Nurse Access** | (28.6%) | 4% | 31.6% | 8.3% | 0% | 12.5% | 25% | 0.75 |
| **Unable to afford missing work for appointments** | (28.6%) | 20% | 26.3% | 33.3% | 0% | 50% | 0% | 0.003 |
| **Unable to afford travel to in-person appointments** | 14.2% | 20% | 5.3% | 8.3% | 0% | 12.5% | 0% | 0.271 |
| **Unable to afford IBD-related medications** | 14.3% | 40% | 36.8% | 16.7% | 25% | 33.3% | 25% | 0.105 |
| **Bought medicine back home** | 0% | 0% | 15.8% | 0% | 0% | 16.7% | 25% | <0.001 |
| **Contacted GI in home country** | 28.6% | 20% | 57.9% | 0% | 0% | 54.2% | 50% | <0.001 |
| **Home food concerns** | 14.3% | 0% | 10.5% | 16.7% | 25% | 20.8% | 0% | <0.001 |
| **Western food concerns** | 42.9% | 40% | 78.9% | 41.7% | 25% | 66.7% | 50% | <0.001 |
| **Ulcerative Colitis (UC)** | 14.3% | 20% | 57.9% | 75% | 0% | 75% | 25% | 0.006 |
| **Crohn's Disease (CD)** | 42.9% | 60% | 42.1% | 8.3% | 50% | 12.5% | 75% | 0.006 |

*Supplemental Table 4*
